# Loss Weightings for Improving Imbalanced Brain Structure Segmentation Using Fully Convolutional Networks

**DOI:** 10.3390/healthcare9080938

**Published:** 2021-07-26

**Authors:** Takaaki Sugino, Toshihiro Kawase, Shinya Onogi, Taichi Kin, Nobuhito Saito, Yoshikazu Nakajima

**Affiliations:** 1 Department of Biomedical Information, Institute of Biomaterials and Bioengineering, Tokyo Medical and Dental University, Tokyo 101-0062, Japan; kawase.bmi@tmd.ac.jp (T.K.); onogi.bmi@tmd.ac.jp (S.O.); 2 Department of Neurosurgery, Graduate School of Medicine, The University of Tokyo, Tokyo 113-0033, Japan; tkin-tky@g.ecc.u-tokyo.ac.jp (T.K.); nsaito-nsu@m.u-tokyo.ac.jp (N.S.)

**Keywords:** brain structure segmentation, fully convolutional networks, class imbalance, loss weighting, magnetic resonance images

## Abstract

Brain structure segmentation on magnetic resonance (MR) images is important for various clinical applications. It has been automatically performed by using fully convolutional networks. However, it suffers from the class imbalance problem. To address this problem, we investigated how loss weighting strategies work for brain structure segmentation tasks with different class imbalance situations on MR images. In this study, we adopted segmentation tasks of the cerebrum, cerebellum, brainstem, and blood vessels from MR cisternography and angiography images as the target segmentation tasks. We used a U-net architecture with cross-entropy and Dice loss functions as a baseline and evaluated the effect of the following loss weighting strategies: inverse frequency weighting, median inverse frequency weighting, focal weighting, distance map-based weighting, and distance penalty term-based weighting. In the experiments, the Dice loss function with focal weighting showed the best performance and had a high average Dice score of 92.8% in the binary-class segmentation tasks, while the cross-entropy loss functions with distance map-based weighting achieved the Dice score of up to 93.1% in the multi-class segmentation tasks. The results suggested that the distance map-based and the focal weightings could boost the performance of cross-entropy and Dice loss functions in class imbalanced segmentation tasks, respectively.

## 1. Introduction

Brain structure segmentation on magnetic resonance (MR) images is an essential technique for measuring, visualizing, and evaluating brain morphology. It is used for diagnosis support of psychiatric and neurodegenerative diseases, brain development analysis, and surgical planning and navigation [1,2]. It is manually performed in practice, but manual segmentation is a very laborious task and is subject to intra- and inter-operator variability [1]. Thus, it is desirable to provide an automatic accurate segmentation of brain structures. The most successful state-of-the-art approach for automated segmentation is a fully convolutional network (FCN) [3]. It enables pixel-wise segmentation in an end-to-end manner. Since it was proposed by Long et al. [3] in 2015, it has been improved for medical image segmentation [4,5] and applied to brain structure segmentation tasks [6]. However, it is often biased towards the majority (large-size) classes and suffers from low segmentation performance on the minority (small-size) classes due to a high imbalance between background and foreground classes in medical images. To address this problem, which is commonly known as the class imbalance, there are two types of approaches: data-level approaches and algorithm-level approaches [7,8].

Data-level approaches mainly alleviate the class imbalance by undersampling the majority classes [9] and oversampling the minority classes [10]. However, the majority undersampling limits the information of available data for training and the minority oversampling can lead to overfitting. On the other hand, algorithm-level approaches address the class imbalance by improving algorithms for training. The most common approach is improving loss functions. The improvement of loss functions can be carried out by using new evaluation metrics for loss function or weighting loss functions to enhance the importance of minority classes in the training process. Thus far, various types of loss functions [11,12,13,14,15,16,17] and loss weighting strategies [4,18,19,20,21,22,23,24,25] have been proposed to alleviate the class imbalance problem. They can be applied for any medical image segmentation tasks in a plug-and-play fashion [26]. However, it is unclear which loss function and weighting strategy should be used in different situations. Thus, it is important to reveal weighted loss functions which can enhance the capability of FCNs in brain structure segmentation tasks.

In related works, Ma et al. [26] performed a systematic study of the utility of 20 loss functions on typical segmentation tasks using public datasets and evaluated the performance of these loss functions in the imbalanced segmentation tasks. Moreover, Ma et al. [27] compared and evaluated the boundary-based loss functions, which minimize the distance between boundaries of ground-truth and predicted segmentation labels, in an empirical study. Yeung et al. [28] focused on compound loss functions, combining Dice and cross-entropy-based losses with a modulating factor of focal loss function [19] and evaluated what compound loss functions were effective to handle class imbalance problems. As shown in these related works, the effect of loss functions varies according to the situation of segmentation tasks (e.g., medical images used for segmentation, the number and size of segmentation target objects, and the degree of class imbalance). However, how the loss functions work for different segmentation targets remains undiscussed, although their accuracies were evaluated in the related works.

We test the effect of weighted loss functions in different situations of imbalanced brain structure segmentation tasks, including binary- and multi-class segmentation tasks. Especially, in this study, we focus on weighting strategies of loss functions, defined based on class frequency, predictive probability, and distance map, and aim to investigate and discuss how the loss weightings affect the performance of FCNs in brain structure segmentation tasks with different class imbalances.

## 2. Materials and Methods

### 2.1. Segmentation Target

**Figure 1:**
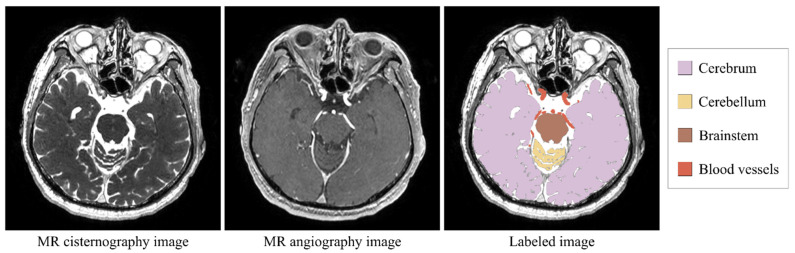
MR images used in this study.

In this study, we adopted a segmentation task of brain structures, including the cerebrum, cerebellum, brainstem, and blood vessels, on MR images. As for MR images, we used MR cisternography (MRC) and MR angiography (MRA) images (Figure 1). MRC images, i.e., heavily T2-weighted images, can clearly represent brain surface and cerebral sulci due to the high intensity of cerebrospinal fluid, whereas MRA images can highlight blood vessels. In our group, we used MRC and MRA as clinical routine MR sequences because of the ease of segmentation processing, and segmented brain parenchyma on MRC images and blood vessels on MRA images for the planning and navigation of neurosurgeries. The brain structures have different features in the MR images. The cerebrum is the largest part of the brain and has a low-level foreground–background imbalance in the MRC images. Its surface, i.e., cerebral sulci, has a bit more of a complex shape. The cerebellum is the second largest part of the brain and is located under the cerebrum. It can be considered a middle-level imbalanced target. The brainstem is a small part of the brain and is located between the cerebrum and the spinal cord. It has a high foreground–background imbalance. The brain parenchyma, i.e., the cerebrum, cerebellum, and brainstem, appears in much the same location in every MRC image volume, although its size and shape have individual differences. Its surface can be clearly visualized in MRC images due to high signal intensity of the cerebrospinal fluid around it. On the other hand, blood vessels have varying locations and shapes and appear as small white spots in MRA images. Thus, they are considered a hard-to-segment target with the high foreground–background imbalance, although they are clearly visualized in MRA images. We used the segmentation targets to fundamentally evaluate the effect of loss weightings on the FCN-based segmentation of different brain structures.

### 2.2. Network Architecture

**Figure 2:**
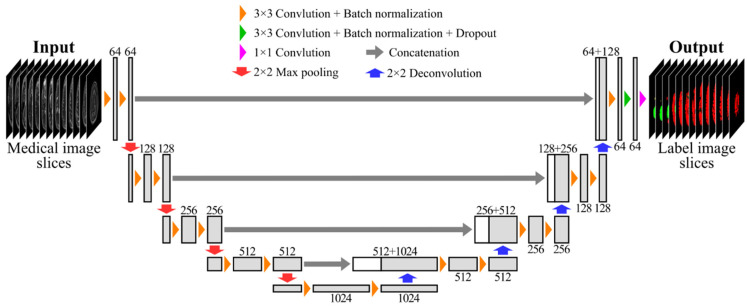
FCN architecture. Each box represents a set of feature maps. The number of feature maps is denoted on the top or bottom of each box.

As an FCN architecture, we adopted a 2D U-net [4], which is one of the most popular FCN architectures for medical image segmentation. Figure 2 shows the network architecture used in this study. The U-net architecture, which consists of a symmetrical encoder–decoder architecture with skip connections, has been often adopted as a baseline FCN architecture for various medical image segmentation tasks. Many different variants of the U-net architecture have been proposed according to different medical image segmentation tasks, and moreover, a 3D U-net architecture [5] has been introduced for volumetric medical image segmentation. However, training the 3D U-net on full input MR image volumes is usually impractical due to memory limitations of the graphical processing unit (GPU). In the case of the MR image volumes used in this study, it would require at least more than 150 GB of GPU memory, which far exceeds the memory of prevalent GPUs. To overcome the memory limitation, approaches to train 3D FCNs on resized or cropped MR image volumes have been proposed. However, resizing MR image volumes to a smaller size may cause the loss of information on segmentation targets, whereas a patch-based approach [5,29] that crops MR image volumes requires the tuning of more hyperparameters (i.e., patch size), which may affect segmentation performance. Thus, in this study, we decided to use the simple 2D U-net architecture to reduce other factors affecting the results as much as possible.

### 2.3. Loss Functions

As shown in the related works [26,27,28], loss functions are an important factor for handling the class imbalance. Existing loss functions for FCN-based segmentation can be divided into four categories: distribution-based loss, region-based loss, boundary-based loss, and compound loss [26]. Distribution-based loss functions measure the dissimilarity between two distributions based on cross-entropy. Region-based loss functions quantify the mismatch or the overlap between two regions. Dice loss function [11,12] is the most common loss function in this category. Boundary-based loss functions measure the distance between two boundaries. Euclidean distance [16] or Hausdorff distance [17] metrics can be used for loss functions in this category. Compound loss functions are defined as the combinations among the distribution-, region-, and boundary-based loss functions [15,28,30,31,32].

As described in [26], most of the distribution-based and region-based loss functions can be considered as the variants of cross-entropy and Dice loss functions, respectively. Moreover, boundary-based loss functions, which are formally defined in a region-based way, have similarities to the Dice loss function. Therefore, as most of the loss functions are based on the cross-entropy and Dice loss functions, we decided to use these two loss functions in this study. The cross-entropy loss $L_{\mathrm{CE}}$ and the Dice loss $L_{\mathrm{Dice}}$ are defined as

(1) $$L_{\mathrm{CE}}=-\frac{1}{N}\overset{C}{\underset{c=1}{\sum }}\overset{N}{\underset{i=1}{\sum }}g_{i,c}\log p_{i,c},$$

(2) $$\begin{array}{l} L_{\mathrm{Dice}}\\ =1-\frac{2\sum_{c=1}^{C}\sum_{i=1}^{N}g_{i,c}p_{i,c}}{2\sum_{c=1}^{C}\sum_{i=1}^{N}g_{i,c}p_{i,c}+\sum_{c=1}^{C}\sum_{i=1}^{N}\left(1-g_{i,c}\right)p_{i,c}+\sum_{c=1}^{C}\sum_{i=1}^{N}g_{i,c}\left(1-p_{i,c}\right)} \end{array}\;=1-\frac{2\sum_{c=1}^{C}\sum_{i=1}^{N}g_{i,c}p_{i,c}}{\sum_{c=1}^{C}\sum_{i=1}^{N}g_{i,c}+\sum_{c=1}^{C}\sum_{i=1}^{N}p_{i,c}},$$

where $g_{i,c}$ and $p_{i,c}$ are the ground-truth label and the predicted segmentation probability of class c at pixel i, respectively. N and C are the numbers of pixels and classes in images for a training dataset, respectively.

### 2.4. Loss Weighting Strategies

In highly imbalanced segmentation tasks, FCNs are likely to ignore small-size foreground classes in the training process, which results in the low segmentation accuracy of the foreground classes. This is what is called the class imbalance problem and can be alleviated by weighting the loss of small-size foreground classes. In this study, we adopted five loss weighting strategies defined based on different factors of class frequency, predictive probability, and distance map. Table 1 indicates the overview of weighted loss functions used in this study. The details of loss weightings are described below.

#### 2.4.1. Inverse Frequency Weighting

Inverse frequency weighting [24], which is one of the most common weighting strategies, is a method for weighting each class based on the class frequency. The weight is inversely proportional to the number of pixels. The smaller the size of target objects is, the higher the weight of them becomes. The inverse frequency weight $W_{c}^{\mathrm{Inverse}}$ in class c is defined by

(3) $$W_{c}^{\mathrm{Inverse}}=\frac{1}{{\left(\sum_{i=1}^{N}g_{i,c}\right)}^{\alpha }},$$

where α is a power parameter. In this study, we used α=1 for the cross-entropy loss function and α=2 for the Dice loss function. The Dice loss function weighted by the inverse of square frequency is known as generalized Dice loss function [24].

#### 2.4.2. Inverse Median Frequency Weighting

Inverse median frequency weighting [18] is a frequency-based weighting as with the inverse frequency weighting. The inverse median frequency weight $W_{c}^{\mathrm{Median}}$ is computed as

(4) $$F_{c}=\frac{\sum_{i=1}^{N}g_{i,c}}{N},$$

(5) $$W_{c}^{\mathrm{Median}}=\frac{median\left(F_{c}\right)}{F_{c}},$$

where $F_{c}$ is the normalized frequency of class c and median(·) denotes a function returning the median value of input data.

#### 2.4.3. Focal Weighting

Focal weighting [19] is a method for putting more focus on hard-to-classify class pixels based on predictive probability. It gives a higher weight to class pixels with lower prediction confidence and reduces the loss assigned to well-classified pixels during the training process. The focal weighting $W_{i,c}^{\mathrm{Focal}}$ is defined by

(6) $$W_{i,c}^{\mathrm{Focal}}={\left(1-p_{i,c}\right)}^{\gamma },$$

where γ is called a focusing parameter. In this study, we used γ=2 for cross-entropy loss function as in [19] and γ=1 for Dice loss function as in [25]. Note that for simplification, here, we did not consider the balancing factor α used in [19].

#### 2.4.4. Distance Transform Map-Based Weighting

**Figure 3:**
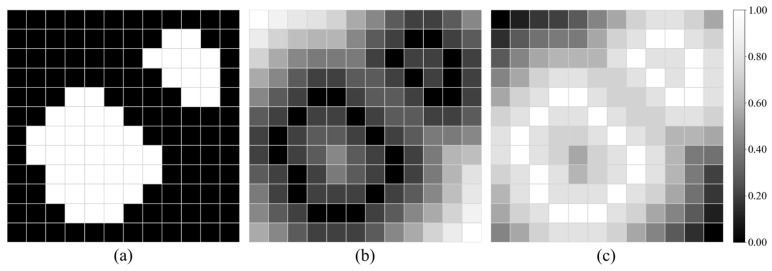
Distance maps for loss weighting. (**a**) Label image, (**b**) distance transform map, and (**c**) distance penalty term.

Distance transform map (DTM), which is computed as the Euclidean distance from the boundary of target objects, is used in the distance-based loss functions [16,17]. Figure 3b shows an example of DTM. DTM-based weighting can be performed by multiplying prediction errors by the DTM. This weighting assigns higher weights to the pixels which are more distant from the boundary of ground-truth labels. Here, we defined the DTM-based weight $W_{c}^{\mathrm{DTM}}$ as

(7) $$DTM_{c}=\{\begin{array}{l} \;\;0,\;\;\;\;\;\;\;\;\;\;\;\;\;\;\;\;\;\;\;\;\;\;\;\;\;x\in \partial G_{c}\\ \underset{y\in \partial G_{c}}{\inf}{\left||x-y|\right|}_{2},\;\;\;\;\;\;\;others \end{array}$$

(8) $$W_{c}^{\mathrm{DTM}}=1+DTM_{c},$$

where $DTM_{c}$ is the distance transform map in class c, and $\partial G_{c}$ denotes the boundary of ground-truth label in class c. ${\left||x-y|\right|}_{2}$ denotes the Euclidean distance between pixels x and y in images.

#### 2.4.5. Distance Penalty Term-Based Weighting

Distance penalty term (DPT) is a distance map for weighting hard-to-segment boundary regions [20], in contrast to the DTM. Let $DPT_{c}$ be the distance penalty term in class c. Then, $DPT_{c}$ is defined as the inverse of the $DTM_{c}$, and thus, it puts higher weights on the pixels closer to the boundary of ground-truth labels in contrast with the DTM-based weighting. Figure 3c shows an example of DPT. As with the DTM-based weighting, DPT-based weighting penalizes prediction errors with the DPT. The DPT-based weight $W_{c}^{\mathrm{DPT}}$ is defined by

(9) $$W_{c}^{\mathrm{DPT}}=1+DPT_{c}.$$

We used the cross-entropy and Dice loss functions weighted by the above five weighting strategies. Table 1 summarizes the weighted loss functions used in this study. As for the weighted Dice loss functions, $L_{\mathrm{Dice}}^{\mathrm{Inverse}}$, $L_{\mathrm{Dice}}^{\mathrm{Median}}$, and $L_{\mathrm{Dice}}^{\mathrm{Focal}}$ put their weights on both the numerator and denominator terms as in [24], while $L_{\mathrm{Dice}}^{\mathrm{DTM}}$ and $L_{\mathrm{Dice}}^{\mathrm{DPT}}$ assign their weights to the false positive (i.e., $\overset{C}{\underset{c=1}{\sum }}\overset{N}{\underset{i=1}{\sum }}\left(1-g_{i,c}\right)p_{i,c}$) and false negative (i.e., $\overset{C}{\underset{c=1}{\sum }}\overset{N}{\underset{i=1}{\sum }}g_{i,c}\left(1-p_{i,c}\right)$) terms in the denominator.

### 2.5. Evaluation of Loss Weighting Strategies

#### 2.5.1. Dataset

We used the MR images of 84 patients with unruptured cerebral aneurysms, which were imaged with MRC and time-of-flight MRA sequences on a 3.0 T scanner (Signa HDxt 3.0 T, GE Healthcare, WI, USA) at the University of Tokyo Hospital, Tokyo, Japan. The MR image volumes had 144–190 slices of 512 × 512 pixels with an in-plane resolution of 0.47 × 0.47 mm^2^ and a slice thickness of 1.00 mm. As a preprocessing step, the MR images were normalized to have a mean of 0 and a standard deviation of 1. The dataset consisting of 84 cases was divided into the following three subsets: training (60 cases), validation (4 cases), and test subsets (20 cases).

The ground-truth-labeled images for training and testing were manually created by using an open-source software for medical image processing (3D Slicer, Brigham and Women’s Hospital, MA, USA); the cerebrum, cerebellum, and brainstem were annotated on MRC images, while blood vessels were annotated on MRA images. The manual annotation was performed by a biomedical engineer and a neurosurgeon. Table 2 indicates the frequency $\left(F_{c}=\overset{N}{\underset{i=1}{\sum }}g_{i,c}/N\right)$ of the foreground classes (the cerebrum, cerebellum, brainstem, and blood vessels) in the training subsets. The cerebrum was the most frequent in the foreground classes, followed by the cerebellum, brainstem, and blood vessels.

#### 2.5.2. Segmentation Tasks

The goal of this work was to study the effect of loss weightings in different class imbalance situations. Thus, we evaluated the effect of loss weightings on both binary- and multi-class segmentation tasks. Table 3 indicates the overview of the training datasets in the binary- and multi-class segmentation tasks.

Binary-class segmentation tasks: To test how the effect of loss weightings varies according to the size of a foreground class in binary-class segmentation tasks, we evaluated the segmentation performance on the binary-class segmentation task for each of the foreground classes. Note that the binary-class segmentation tasks for the cerebrum, cerebellum, and brainstem were performed using MRC images, whereas the binary-class segmentation for blood vessels was performed using MRA images.

Multi-class segmentation tasks: To test how the effect of loss weightings varies according to the imbalance of foreground classes in multi-class segmentation tasks, we evaluated the segmentation performance on the three-, four-, and five-class segmentation tasks; the three, four, and five classes include the foreground classes of (cerebrum, blood vessels), (cerebrum, cerebellum, blood vessels), and (cerebrum, cerebellum, brainstem, blood vessels), respectively. Note that the multi-class segmentation tasks were performed using multi-modal MR images which included MRC and MRA images.

#### 2.5.3. Network Training Procedure

In the binary- and multi-class segmentation tasks, we trained the FCN model on each training dataset using the cross-entropy and Dice loss functions with or without the loss weightings. The FCN model was trained from scratch for 30 epochs with the Adam optimization algorithm [33] (α (learning rate)={1e−3, 1e−4, and 1e−5}, $\beta_{1}=0.9$, $\beta_{2}=0.999$, and epsilon=1e−7) and a batch size of 5 in each training process. For testing, we used the best trained model in the set {learning rate, epoch}={1e−3, 10}, {1e−3, 20}, {1e−3, 30}, {1e−4, 10}, {1e−4, 20}, {1e−4, 30}, {1e−5, 10}, {1e−5, 20}, and {1e−5, 30} because the condition for good training convergence, especially learning rate and number of epochs, was different according to the loss weightings.

The FCN model with the weighted loss functions were implemented by using Keras with Tensorflow backend, and the training and prediction were performed on an Ubuntu 16.04 PC (CPU: Intel Xeon Gold 5222 3.80 GHz, RAM: 384 GB) with NVIDIA Quadro RTX8000 GPU cards for deep learning.

#### 2.5.4. Evaluation Metrics

To quantitatively evaluate the segmentation performance, we adopted the Dice similarity coefficient (DSC), surface DSC (SDSC) [34], average symmetric surface distance (ASD), and Hausdorff distance (HD). The DSC and SDSC, overlap-based metrics, can be used for evaluating the region overlaps; the DSC measures the overlap of whole regions between ground-truth and predicted labels, whereas the SDSC measures the overlap of the two surface regions. The DSC was calculated by

(10) $$\mathrm{DSC}=\frac{2\left|G\cap P\right|}{\left|G\right|+\left|P\right|},$$

where G and P denote the regions of ground-truth and predicted labels, respectively. The SDSC was calculated by

(11) $$\mathrm{SDSC}=\frac{\left|\partial G\cap B_{\partial P}^{\left(\tau \right)}\right|+\left|\partial P\cap B_{\partial G}^{\left(\tau \right)}\right|}{\left|\partial G\right|+\left|\partial P\right|},$$

where ∂G and ∂P denote the boundaries of ground-truth and predicted labels, respectively. $B_{\partial G}^{\left(\tau \right)},B_{\partial P}^{\left(\tau \right)}\subset {\mathbb{R}}^{3}$ are the border regions of ground-truth and predicted label surfaces at tolerance τ, which are defined as $B_{\partial G}^{\left(\tau \right)}=\left\{x\in {\mathbb{R}}^{3}|\exists y\in \partial G,\;||x-y||\leq \tau \right\}$ and $B_{\partial P}^{\left(\tau \right)}=\left\{x\in {\mathbb{R}}^{3}|\exists y\in \partial P,\;||x-y||\leq \tau \right\}$, respectively [26,34]. We here used τ=1 mm as in [26].

The ASD and HD, boundary distance-based metrics, can be used for evaluating the surface errors; ASD measures the average surface distance between ground-truth and predicted labels, whereas HD measures the max surface distance between them. The ASD was calculated by

(12) $$\mathrm{ASD}=\frac{\sum_{x\in \partial G}D\left(x,\partial P\right)+\sum_{y\in \partial G}D\left(y,\partial G\right)}{\left|\partial G\right|+\left|\partial P\right|},$$

where D(a,A) denote the minimum Euclidean distance from a voxel a to a set of voxels A. The HD was calculated by

(13) $$\mathrm{HD}=\underset{}{\max}\left\{\underset{x\in \partial G}{\max}D\left(x,\partial P\right),\;\underset{y\in \partial P}{\max}D\left(y,\partial G\right)\right\}.$$

As for HD, in this study, 95th-percentile HD (95HD) was used, as in [27].

When the segmentation accuracy increases, the overlap-based and the boundary distance-based metrics approach 1 and 0, respectively. The evaluation metrics was implemented using the open-source code, which is available at [35].

Furthermore, we used a rank score, which was defined based on [36], to comprehensively evaluate which loss weightings worked well based on the above metrics, as in [26]. The rank score was computed according to the following steps:

Step 1. Performance assessment per case: compute metrics $m_{i}\left(loss_{j},class_{k},\;case_{l}\right)\;\left(i=1,\ldots ,\;N_{m}\right)$ of all loss functions $loss_{j}\;\left(j=1,\ldots ,\;12\right)$ for all classes $class_{k}\;\left(k=1,\ldots ,\;N_{c}\right)$ in all test cases ${case}_{l}\;\left(l=1,\ldots ,\;20\right)$, where $N_{m}$ and $N_{c}$ are the number of metrics and classes, respectively. Note that in this case, we used four metrics $m_{i}\in \left\{\mathrm{DSC},\;\mathrm{SDSC},\;\mathrm{ASD},\;95\mathrm{HD}\right\}$ and a total of twelve loss functions, including cross-entropy and Dice loss functions with no weighting, Inverse, Median, Focal, DTM, and DPT weightings.
Step 2. Statistical tests: perform Wilcoxon signed-rank pairwise statistical tests between all loss functions with the values $m_{i}\left(loss_{j},class_{k},\;case_{l}\right)-m_{i}\left(loss_{j}^{\prime },class_{k},\;case_{l}\right)$.
Step 3. Significance scoring: compute a significance score $s_{ik}\left(loss_{j}\right)$ for loss functions $loss_{j}$, classes $class_{k}$, and metrics $m_{i}$. $s_{ik}\left(loss_{j}\right)$ equals the number of loss functions performing significantly worse than $loss_{j}$ according to the statistical tests (p<0.05, not adjusted for multiplicity).
Step 4. Rank score computing: compute the final rank score $R\left(loss_{j}\right)$ of each loss function from the mean significance score of all classes and metrics in each of the binary- and multi-class segmentation tasks by the following equation:
  (14) $$R\left(loss_{j}\right)=\frac{1}{N_{m}\times N_{c}}\overset{N_{m}}{\underset{i=1}{\sum }}\overset{N_{c}}{\underset{k=1}{\sum }}s_{ik}\left(loss_{j}\right).$$

## 3. Results

We compared the results of loss weightings (inverse frequency weighting (Inverse), inverse median frequency weighting (Median), focal weighting (Focal), distance transform map-based weighting (DTM), and distance penalty term-based weighting (DPT)) with those of no weighting (N/A). The statistical difference between N/A and each loss weighting was evaluated by the Wilcoxon signed-rank test. A *p*-value less than 0.05 was considered significant. Subsequently, we comprehensively evaluated the effect of loss weightings by using the rank scores.

### 3.1. Binary-Class Segmentation Tasks

**Figure 4:**
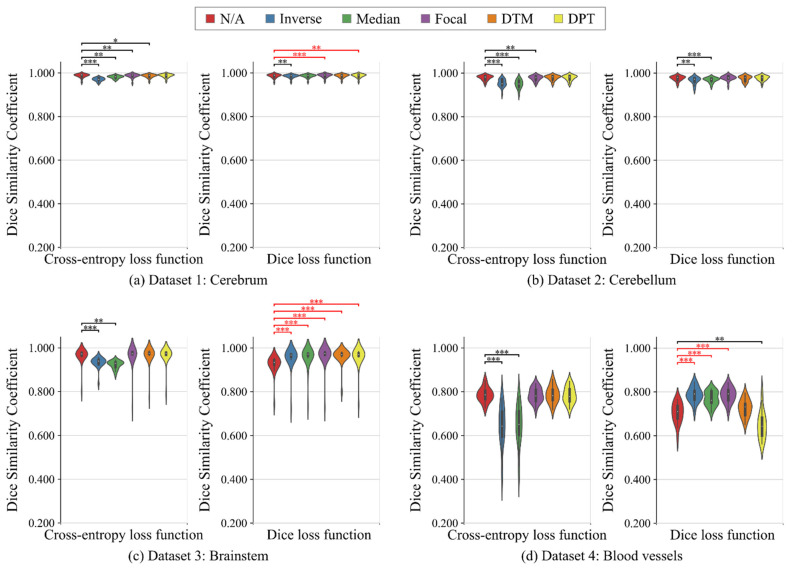
Violin plots of the segmentation results (Dice similarity coefficients) of no weighting (N/A), inverse frequency weighting (Inverse), inverse median frequency weighting (Median), focal weighting (Focal), distance transform map-based weighting (DTM), and distance penalty term-based weighting (DPT) in binary-class segmentation tasks. (**a**) Dataset 1: cerebrum, (**b**) Dataset 2: cerebellum, (**c**) Dataset 3: brainstem, and (**d**) Dataset 4: blood vessels. Compared with the results of N/A, the significantly worse and better results are shown in black and red, respectively (Wilcoxon signed-rank test, *  p<0.05, **  p<0.01, and ***  p<0.001, not adjusted for multiplicity).

Table 4 summarizes all the results in the binary-class segmentation tasks. Figure 4 shows the violin plots of the Dice scores. As for cross-entropy loss function, Inverse and Median provided worse results than N/A in any segmentation tasks. Focal, DTM, and DPT tended to improve the surface accuracy in the highly imbalanced segmentation tasks (i.e., segmentation of brainstem and blood vessels) although the improvement was not statistically significant. As for Dice loss function, Inverse and Median significantly improved the segmentation accuracy in the highly imbalanced segmentation tasks, compared with N/A. Focal tended to provide better results than N/A in all the binary-class segmentation tasks. The distance map-based weightings (i.e., DTM and DPT) worked well in the segmentation of brain parenchyma, but they were ineffective in the segmentation of blood vessels.

**Figure 5:**
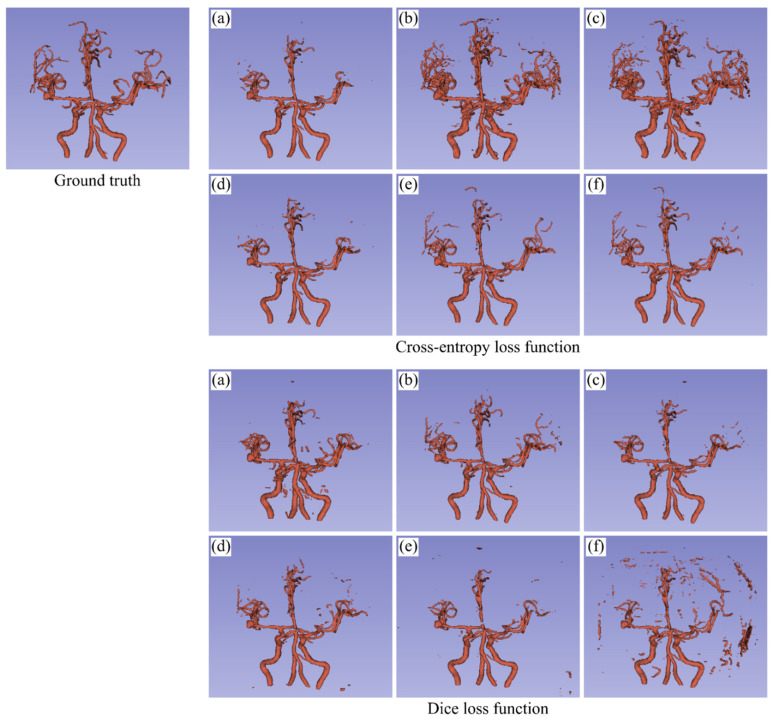
Visualization of the segmentation results of blood vessels in the binary-class segmentation task. (**a**) No weighting, (**b**) Inverse frequency weighting, (**c**) Inverse median frequency weighting, (**d**) Focal weighting, (**e**) Distance transform map-based weighting, and (**f**) Distance penalty term-based weighting.

Figure 5 visualizes an example of the segmentation results of blood vessels, which are the highly imbalanced class, in the binary-class segmentation task. As for the cross-entropy loss function, N/A had difficulty in segmenting the upper blood vessels. Both Inverse and Median allowed the FCN to extract most of the upper blood vessels which N/A failed to segment, but obviously increased the overextraction. Focal provided almost the same result as N/A. Both DTM and DPT extracted the wider region of blood vessels than N/A. As for the Dice loss function, N/A had false negatives in the upper blood vessels as with the cross-entropy loss function. It also provided a few more false positives. The class frequency-based weightings, especially Inverse, improved the false positives as well as the false negatives. Focal provided better results than N/A, although it was not so much as Inverse. The results of the distance map-based weightings, especially DPT, were worse than that of N/A.

### 3.2. Multi-Class Segmentation Tasks

**Figure 6:**
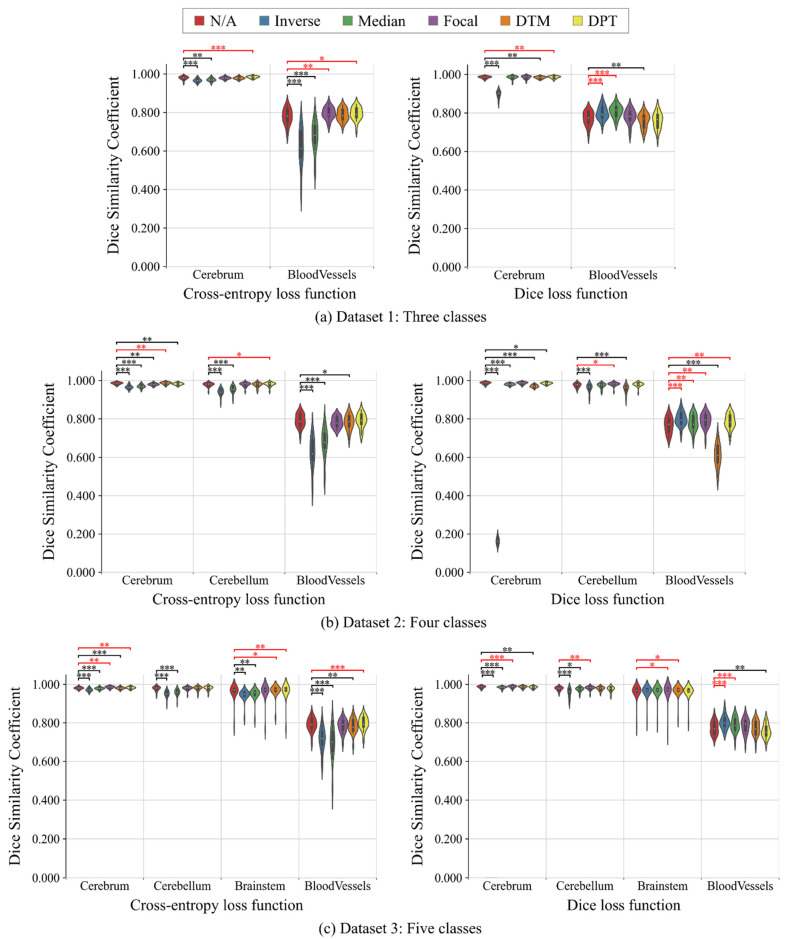
Violin plots of the segmentation results (Dice similarity coefficients) of no weighting (N/A), inverse frequency weighting (Inverse), inverse median frequency weighting (Median), focal weighting (Focal), distance transform map-based weighting (DTM), and distance penalty term-based weighting (DPT) in multi-class segmentation tasks. (**a**) Dataset 1: three classes, (**b**) Dataset 2: four classes, and (**c**) Dataset 3: five classes. Compared with the results of N/A, the significantly worse and better results are shown in black and red, respectively (Wilcoxon signed-rank test, *  p<0.05, **  p<0.01, and ***  p<0.001, not adjusted for multiplicity).

Table 5 summarizes all the results in the multi-class segmentation tasks. Figure 6 shows the violin plots of the Dice scores. As for the cross-entropy loss function, Inverse and Median, as in the binary-class segmentation tasks, worsened the results in any multi-class segmentation tasks. The results of Focal, especially surface accuracies, were equivalent to or better than those of N/A in almost all the tasks. In the distance map-based weighting, DPT worked well for improvement of segmentation accuracy. As for the Dice loss function, Inverse and Median significantly improved the segmentation accuracy of blood vessels, which were a very high-level imbalanced class, in any multi-class segmentation tasks. However, Inverse also significantly worsened the segmentation accuracy of the cerebrum and cerebellum, which were relatively large-size targets. Focal provided better results than N/A for almost all the segmentation targets. The distance map-based weightings showed inconsistent results between the multi-class segmentation tasks.

**Figure 7:**
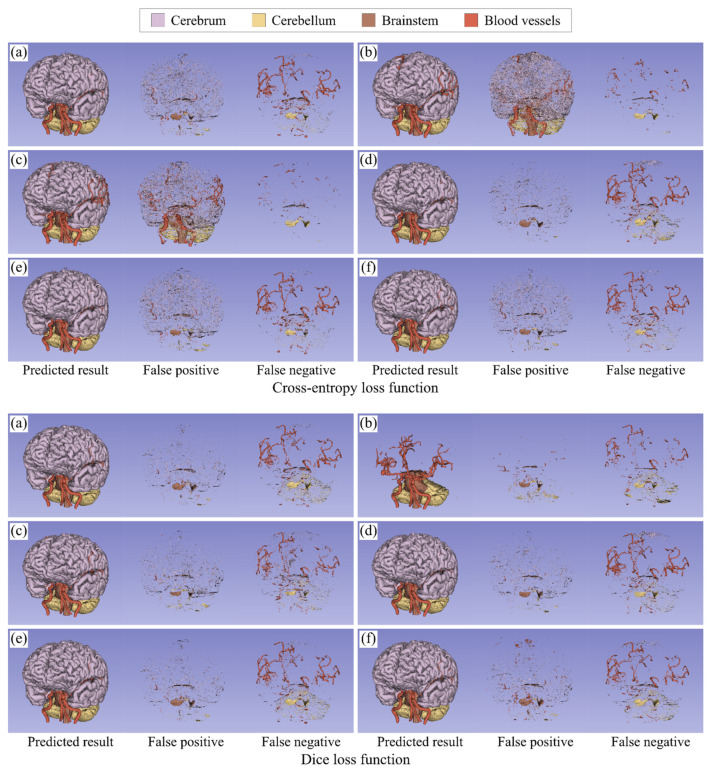
Visualization of the segmentation results in the five-class segmentation task. (**a**) No weighting, (**b**) inverse frequency weighting, (**c**) inverse median frequency weighting, (**d**) focal weighting, (**e**) distance transform map-based weighting, and (**f**) distance penalty term-based weighting. The segmentation results include the predicted results (left), the false positives (middle), and the false negatives (right). Note that in the result of Dice loss function with inverse frequency weighting, there are no true positive voxels in the cerebrum class and most of the background region were overestimated as the cerebrum class, but the false positives and false negatives in the cerebrum class were excluded from the figure for better visualization.

Figure 7 visualizes an example of the segmentation results in the five-class segmentation task. It shows the false positive and false negative labels as well as the predicted labels. False positives were likely to appear around the surface of the cerebrum, cerebellum, and brainstem, while false negatives tended to appear in the upper part of blood vessels. As for the cross-entropy loss function, Inverse and Median reduced the false negatives, but more than that, they greatly increased the false positives. Focal worked well for a reduction in the false positives, although it did not reduce the false negatives. The results of the distance map-based weightings showed that DPT was a little effective in reducing the false positives and false negatives. As for Dice loss function, Inverse reduced the false negatives in blood vessels, although it failed to segment the whole cerebrum. Median worked to reduce the false negatives in blood vessels, as with Inverse. Focal slightly reduced the false positives. DTM and DPT seemed to provide almost the same results as N/A.

### 3.3. Rank Scoring

Table 6 indicates the ranking results of loss weightings in the binary- and multi-class segmentation tasks. The distance map-based weightings for cross-entropy loss function and the predictive-probability weighting for Dice loss function tended to have high rank scores in both the binary- and multi-class segmentation tasks. In the binary-class segmentation tasks, the Dice loss function with Focal showed the best ranking result. It actually obtained a high average DSC and SDSC of 92.8% and 93.3%, respectively. Compared with no weighting, it improved the DSC and SDSC values of all tasks by 0.2–8.1% and 0.5–12.5%, respectively. In the multi-class segmentation tasks, the cross-entropy loss function with DPT had the highest rank score, followed by the Dice loss function with Focal. In the five-class segmentation task, DPT achieved the highest average DSC and SDSC values of 93.1% and 94.6%, respectively.

## 4. Discussion

We evaluated the effect of loss weightings on the segmentation of the cerebrum, cerebellum, brainstem, and blood vessels from the MR images. From the segmentation results with the non-weighted loss functions, we found that the segmentation errors of the cerebrum, cerebellum, and brainstem, including false positives and false negatives, were concentrated at the edges of them, whereas the segmentation errors of blood vessels, especially false negatives, appeared in the upper part of them. This is probably because the edges of brain parenchyma or the upper blood vessels were variable according to the cases and the FCN was biased toward training image features on easier-to-segment majority regions. Thus, in order to improve the brain structure segmentation, it would be important to make the FCN focus on training image features around the edge of brain parenchyma and in the upper part of blood vessels by loss weightings. We discuss the effect of loss weightings based on the results in the binary- and multi-class segmentation tasks below. Subsequently, we also discuss the limitations of this study.

### 4.1. Binary-Class Segmentation Tasks

As for the cross-entropy loss function, the class frequency-based weightings (Inverse and Median) greatly increased false positives. They assign a lower uniform weight to the loss of larger-size classes, i.e., background class in the case of binary-class segmentation tasks. They gave a low uniform weight to low-confidence background pixels near the edge of the foreground, which would result in a large increase in false positives on the low-confidence background pixels, although they could also help reduce false negatives. On the other hand, the predictive probability- and the distance map-based weightings tended to improve the surface accuracy of highly imbalanced classes, i.e., the brainstem and blood vessels. Different from the class frequency-based weighting, they assign a different weight to each pixel. Using such pixel-wise weights instead of uniform weights may be appropriate for imbalanced segmentation because FCNs do not focus equally on all the pixels of the same class during training. The predictive-probability-based weighting (Focal) gives higher weights to pixels with lower prediction confidences based on the predictive probability and helps correct pixels misclassified with low prediction confidence, whereas the distance map-based weightings (DTM and DPT) define pixel-wise weights based on the distance from the edge of ground-truth labels and help correct surface segmentation errors. Thus, it is considered that these loss weightings could correct the surface error because pixels around the edge of foreground class were subject to be misclassified with low prediction confidence in the highly imbalanced segmentation tasks.

As for the Dice loss function, the class frequency-based weightings significantly improved the accuracy in the highly imbalanced segmentation tasks, although they did not work well for the cross-entropy loss function. They assigned the weight to both the denominator and numerator for the Dice loss function, which would allow the FCN to reduce false negatives without increasing false positives. The predictive probability-based weighting, which showed the best performance in Table 6, worked well for the low- and middle-level imbalanced segmentation tasks as well as the highly imbalanced segmentation tasks. This can be explained by the fact that the FCN with the Dice loss function had more pixels misclassified with low prediction confidence in the low- and middle-level imbalanced segmentation tasks, compared with that of the cross-entropy loss function. Additionally, the distance map-based weightings tended to improve the surface accuracy in the brain parenchyma segmentation. However, they were ineffective in the segmentation task of blood vessels. As shown in [16], in the case of the segmentation of objects which have variable locations and shapes, they might be able to work stably by using a scheduling strategy, i.e., gradually increasing the weight to the mismatched region with the training epochs.

### 4.2. Multi-Class Segmentation Tasks

The binary-class segmentation tasks included the class imbalance problem between background and foreground classes, whereas the multi-class segmentation tasks, which deal with two or more foreground classes, included the class imbalance problems not only between background and foreground classes but also among foreground classes. However, the results in the multi-class segmentation tasks showed similar tendencies to those in the binary-class segmentation tasks, although some of them were affected by the foreground–foreground class imbalance.

The class frequency-based weightings failed to improve the segmentation performance of the FCN with the cross-entropy loss function in any multi-class segmentation tasks because they greatly increased false positives by assigning an extremely low weight to the background pixels. For the Dice loss function, they also worked negatively for the low- and middle-level imbalanced classes. Especially in the five-class segmentation task, Inverse could not segment the cerebrum at all due to the foreground–foreground class imbalance. However, it also provided the best DSC value for blood vessels. Thus, the class frequency-based weightings could work well for only objects with very high imbalance because of their extreme weighting in any segmentation tasks. The predictive probability-based weighting totally worked well for both the cross-entropy and Dice loss functions. These results suggested that despite the foreground–foreground class imbalance, it could enable FCNs to focus on the pixels misclassified with low prediction confidence, i.e., hard-to-segment pixels, by considering the predictive probability. As well, the distance map-based weightings tended to provide good segmentation results for the cross-entropy loss function. In particular, the cross-entropy loss function with DPT achieved the best performance as indicated in Table 6b. However, the distance map-based weightings provided unstable segmentation results for the Dice loss function. In this study, although we designed the Dice loss function with the distance map-based weightings by multiplying the false positive and false negative terms in the denominator by the weights, using a scheduling strategy might make the effect of the distance map-based weightings more stable, as mentioned above.

Therefore, the cross-entropy loss function with DPT and the Dice loss function with Focal achieved relatively high accuracy in any segmentation targets and tasks, but some other weightings outperformed their weightings according to segmentation targets. For example, the Dice loss function with Inverse provided better DSC and SDSC results for blood vessels than that with Focal. Therefore, in this study, we focused on the unary weighted loss functions instead of compound loss functions, but considering the difference of features in loss weightings, the combination of different weighted loss functions might lead to the further improvement of segmentation performance.

### 4.3. Limitations

For limitations of this work, we adopted the segmentation of brain parenchyma and blood vessels on MRC and MRA images, which is performed as a routine work in our group. However, the effect of loss weightings might depend on segmentation targets and tasks, although the results in this study reflected the features of loss weightings. Considering a wider range of applications, we should test the loss weightings in other brain structure segmentation tasks (e.g., the segmentation of white matter, gray matter, and cerebrospinal fluid on T1-weighted MR images). Second, we used the 2D U-Net architecture to investigate the effect of loss weightings with less hyperparameters. However, we would need to test 3D FCNs with the weighted loss functions, because they have been applied for volumetric brain structure segmentation. Moreover, we set default parameters for loss weightings (e.g., the focusing parameter for focal weighting) based on the previous studies, but tuning such parameters would enable the performance improvement of FCNs. Furthermore, in this study, we focused on segmenting brain structures, including blood vessels, from the MR images of patients with cerebral aneurysms, but considering the clinical practice, it would be desired to automatically detect the location of aneurysms, as in [37], in addition to the segmentation.

## 5. Conclusions

This paper investigated how the loss weightings work for FCN-based brain structure segmentation on MR images in different class imbalance situations. Using the 2D U-Net with cross-entropy or Dice loss functions as a baseline network, we tested the five loss weightings, which were defined based on class frequency, predictive probability, and distance map, in the binary- and multi-class brain structure segmentation on MRC and MRA images. From the experimental results, we found that the cross-entropy loss function with the distance map-based weightings, especially distance penalty term-based weighting, and the Dice loss function with the predictive probability-based weighting could stably provide good segmentation results. In the binary-class segmentation tasks, the Dice loss function with focal weighting showed the best performance and achieved a high average DSC of 92.8%, whereas in the multi-class segmentation tasks, the cross-entropy loss function with distance penalty term-based weighting provided the best performance. It achieved the highest average DSC of 93.1% in the five-class segmentation task. We also found that their weighted loss functions were relatively robust to the foreground–foreground class imbalance as well as the background–foreground class imbalance. In other words, the experimental results suggested that they could work well in the situations of both binary- and multi-class segmentation. Therefore, it may be effective to use the distance penalty term-based weighting in the cross-entropy loss function and the focal weighting in the Dice loss function. We believe that these findings would help to select weighting strategies for loss functions or design advanced loss weighting strategies.

In future work, for clinical application, we will address the detection and segmentation of a diseased area that is more highly imbalanced, such as a cerebral aneurysm, as well as its surrounding structures, by using the loss weighting strategies. Moreover, we will design compound loss functions (i.e., combination among the loss weightings) and further investigate the effect of them for different brain structure segmentation tasks.

## Figures and Tables

**Table 1:** Overview of the weighted loss functions.

Baseline Loss Functions	Weighting Strategies		Weighted Loss Functions
Cross-entropy loss function $L_{\mathrm{CE}}$	Class frequency-based weighting	Inverse frequency weighting	$L_{\mathrm{CE}}^{\mathrm{Inverse}}=-\frac{1}{N}\overset{C}{\underset{c=1}{\sum }}W_{c}^{\mathrm{Inverse}}\overset{N}{\underset{i=1}{\sum }}g_{i,c}\log p_{i,c}$
		Inverse median weighting	$L_{\mathrm{CE}}^{\mathrm{Median}}=-\frac{1}{N}\overset{C}{\underset{c=1}{\sum }}W_{c}^{\mathrm{Median}}\overset{N}{\underset{i=1}{\sum }}g_{i,c}\log p_{i,c}$
	Predictive probability-based weighting	Focal weighting	$L_{\mathrm{CE}}^{\mathrm{Focal}}=-\frac{1}{N}\overset{C}{\underset{c=1}{\sum }}\overset{N}{\underset{i=1}{\sum }}W_{i,c}^{\mathrm{Focal}}g_{i,c}\log p_{i,c}$
	Distance map-based weighting	Distance transform map-based weighting	$L_{\mathrm{CE}}^{\mathrm{DTM}}=-\frac{1}{N}\overset{C}{\underset{c=1}{\sum }}\overset{N}{\underset{i=1}{\sum }}W_{c}^{\mathrm{DTM}}g_{i,c}\log p_{i,c}$
		Distance penalty term-based weighting	$L_{\mathrm{CE}}^{\mathrm{DPT}}=-\frac{1}{N}\overset{C}{\underset{c=1}{\sum }}\overset{N}{\underset{i=1}{\sum }}W_{c}^{\mathrm{DPT}}g_{i,c}\log p_{i,c}$
Dice loss function $L_{\mathrm{Dice}}$	Class frequency-based weighting	Inverse frequency weighting	$L_{\mathrm{Dice}}^{\mathrm{Inverse}}=1-\frac{2\sum_{c=1}^{C}W_{c}^{\mathrm{Inverse}}\sum_{i=1}^{N}g_{i,c}p_{i,c}}{\sum_{c=1}^{C}W_{c}^{\mathrm{Inverse}}\sum_{i=1}^{N}\left(g_{i,c}+p_{i,c}\right)}$
		Inverse median weighting	$L_{\mathrm{Dice}}^{\mathrm{Median}}=1-\frac{2\sum_{c=1}^{C}W_{c}^{\mathrm{Median}}\sum_{i=1}^{N}g_{i,c}p_{i,c}}{\sum_{c=1}^{C}W_{c}^{\mathrm{Median}}\sum_{i=1}^{N}\left(g_{i,c}+p_{i,c}\right)}$
	Predictive probability-based weighting	Focal weighting	$L_{\mathrm{Dice}}^{\mathrm{Focal}}=1-\frac{2\sum_{c=1}^{C}\sum_{i=1}^{N}W_{i,c}^{\mathrm{Focal}}g_{i,c}p_{i,c}}{\sum_{c=1}^{C}\sum_{i=1}^{N}W_{i,c}^{\mathrm{Focal}}\left(g_{i,c}+p_{i,c}\right)}$
	Distance map-based weighting	Distance transform map-based weighting	$L_{\mathrm{Dice}}^{\mathrm{DTM}}=1-\left(2\overset{C}{\underset{c=1}{\sum }}\overset{N}{\underset{i=1}{\sum }}g_{i,c}p_{i,c}\right)/\left(2\overset{C}{\underset{c=1}{\sum }}\overset{N}{\underset{i=1}{\sum }}g_{i,c}p_{i,c}+\overset{C}{\underset{c=1}{\sum }}\overset{N}{\underset{i=1}{\sum }}W_{c}^{\mathrm{DTM}}\left(1-g_{i,c}\right)p_{i,c}+\overset{C}{\underset{c=1}{\sum }}\overset{N}{\underset{i=1}{\sum }}W_{c}^{\mathrm{DTM}}g_{i,c}\left(1-p_{i,c}\right)\right)$
		Distance penalty term-based weighting	$L_{\mathrm{Dice}}^{\mathrm{DPT}}=1-\left(2\overset{C}{\underset{c=1}{\sum }}\overset{N}{\underset{i=1}{\sum }}g_{i,c}p_{i,c}\right)/\left(2\overset{C}{\underset{c=1}{\sum }}\overset{N}{\underset{i=1}{\sum }}g_{i,c}p_{i,c}+\overset{C}{\underset{c=1}{\sum }}\overset{N}{\underset{i=1}{\sum }}W_{c}^{\mathrm{DPT}}\left(1-g_{i,c}\right)p_{i,c}+\overset{C}{\underset{c=1}{\sum }}\overset{N}{\underset{i=1}{\sum }}W_{c}^{\mathrm{DPT}}g_{i,c}\left(1-p_{i,c}\right)\right)$

**Table 2:** Frequency of the foreground classes in the training subset (n=60).

	Cerebrum	Cerebellum	Brainstem	Blood Vessels
Frequency	0.096	0.012	0.003	0.001

**Table 3:** Training datasets in binary- and multi-class segmentation tasks. BG, CR, CL, BS, and BV stand for background, cerebrum, cerebellum, brainstem, and blood vessels, respectively.

Dataset	Ratio ^1^
Binary-class segmentation tasks	
Dataset 1: Cerebrum	BG:CR=9:1
Dataset 2: Cerebellum	BG:CL=86:1
Dataset 3: Brainstem	BG:BS=352:1
Dataset 4: Blood vessels	BG:BV=749:1
Multi-class segmentation tasks	
Dataset 1: Three classes	BG:CR:BV=677:72:1
Dataset 2: Four classes	BG:CR:CL:BV=668:72:9:1
Dataset 3: Five classes	BG:CR:CL:BS:BV=666:72:9:2:1

^1^ Ratio of the number of labeled voxels between foreground classes in each training dataset.

**Table 4:** Segmentation results of no weighting (N/A), inverse frequency weighting (Inverse), inverse median frequency weighting (Median), focal weighting (Focal), distance transform map-based weighting (DTM), and distance penalty term-based weighting (DPT) in binary-class segmentation tasks: Dice similarity coefficient (DSC), surface DSC (SDSC), average symmetric surface distance (ASD) (mm), and 95th-percentile Hausdorff distance (95HD) (mm). (a) Dataset 1: cerebrum, (b) Dataset 2: cerebellum, (c) Dataset 3: brainstem, and (d) Dataset 4: blood vessels. The results of background class are excluded in this table. Compared with the results of N/A, the significantly better and worse results are shown in bold and italic, respectively (Wilcoxon signed-rank test, p<0.05, not adjusted for multiplicity).

Loss Function	Weighting	DSC	SDSC	ASD	95HD
**(a) Dataset 1: Cerebrum**					
Cross entropy	N/A	0.987	0.991	0.064	0.287
	Inverse	*0.970*	*0.941*	*0.424*	*3.504*
	Median	*0.981*	*0.983*	*0.135*	*0.565*
	Focal	*0.986*	*0.989*	*0.073*	*0.397*
	DTM	*0.986*	0.990	0.069	0.378
	DPT	0.987	**0.992**	0.059	0.328
Dice	N/A	0.986	0.988	0.102	0.381
	Inverse	*0.984*	0.986	*0.275*	0.495
	Median	0.985	0.990	*0.234*	0.425
	Focal	**0.988**	**0.993**	**0.054**	0.308
	DTM	0.987	**0.991**	**0.061**	0.364
	DPT	**0.987**	**0.992**	**0.066**	0.341
**(b) Dataset 2: Cerebellum**					
Cross entropy	N/A	0.978	0.981	0.088	0.669
	Inverse	*0.954*	*0.922*	*0.411*	*1.755*
	Median	*0.950*	*0.904*	*0.525*	*2.539*
	Focal	*0.976*	*0.976*	0.166	*2.430*
	DTM	0.978	*0.978*	0.104	0.729
	DPT	0.978	0.980	0.089	0.713
Dice	N/A	0.976	0.973	0.221	1.048
	Inverse	*0.965*	*0.940*	*1.934*	*1.975*
	Median	*0.968*	*0.950*	*2.037*	*4.568*
	Focal	0.977	**0.980**	**0.101**	0.686
	DTM	0.974	0.972	0.153	0.878
	DPT	0.976	0.975	**0.184**	2.331
**(c) Dataset 3: Brainstem**					
Cross entropy	N/A	0.963	0.940	0.501	4.676
	Inverse	*0.933*	*0.874*	*1.024*	*8.518*
	Median	*0.922*	*0.849*	*0.849*	*6.510*
	Focal	0.962	0.947	**0.239**	1.362
	DTM	0.965	0.951	0.280	**1.204**
	DPT	0.965	0.946	0.425	3.478
Dice	N/A	0.923	0.824	8.880	156.912
	Inverse	**0.953**	**0.921**	**0.476**	**4.770**
	Median	**0.954**	**0.926**	**0.421**	**3.365**
	Focal	**0.963**	**0.949**	**0.241**	**1.905**
	DTM	**0.961**	**0.939**	**0.332**	**4.268**
	DPT	**0.957**	**0.936**	**0.318**	**1.646**
**(d) Dataset 4: Blood vessels**					
Cross entropy	N/A	0.785	0.809	1.415	12.947
	Inverse	*0.642*	*0.700*	*2.008*	*16.978*
	Median	*0.647*	*0.690*	*2.222*	*18.620*
	Focal	0.783	0.812	1.351	12.353
	DTM	0.786	0.821	1.419	12.243
	DPT	0.784	0.824	1.361	12.340
Dice	N/A	0.704	0.767	1.996	16.026
	Inverse	**0.786**	**0.826**	**1.385**	**13.364**
	Median	**0.768**	**0.794**	**1.627**	14.597
	Focal	**0.785**	**0.812**	**1.518**	13.104
	DTM	0.725	0.754	*2.400*	*19.281*
	DPT	*0.648*	*0.627*	*5.999*	*40.077*

**Table 5:** Segmentation results of no weighting (N/A), inverse frequency weighting (Inverse), inverse median frequency weighting (Median), focal weighting (Focal), distance transform map-based weighting (DTM), and distance penalty term-based weighting (DPT) in the multi-class segmentation tasks: Dice similarity coefficient (DSC), surface DSC (SDSC), average symmetric surface distance (ASD), and 95th-percentile Hausdorff distance (95HD). (a) Dataset 1: three classes, (b) Dataset 2: four classes, and (c) Dataset 3: five classes. The results of background class are excluded in this table. Compared with the results of N/A, the significantly better and worse results are shown in bold and italic, respectively (Wilcoxon signed-rank test, p<0.05, not adjusted for multiplicity).

(a) Dataset 1: Three Classes													
Loss Function	Weighting	Cerebrum								Blood Vessels			
		DSC		SDSC		ASD		95HD		DSC	SDSC	ASD	95HD
Cross entropy	N/A	0.979		0.965		0.507		5.635		0.778	0.810	1.926	17.142
	Inverse	*0.967*		0.956		0.265		1.256		*0.618*	*0.662*	2.448	20.272
	Median	*0.970*		0.969		0.239		1.273		*0.675*	*0.740*	1.901	17.298
	Focal	0.979		0.989		0.093		0.585		**0.796**	**0.843**	**1.195**	12.933
	DTM	0.979		0.989		0.092		0.585		0.788	**0.848**	**1.097**	**10.539**
	DPT	**0.984**		**0.992**		**0.069**		**0.492**		**0.795**	**0.836**	**1.198**	**11.321**
Dice	N/A	0.985		0.990		0.266		0.445		0.771	0.833	1.225	11.276
	Inverse	*0.896*		*0.634*		*2.290*		*17.436*		**0.800**	0.842	1.177	11.325
	Median	0.985		*0.986*		**0.109**		0.479		**0.809**	**0.848**	1.172	11.654
	Focal	0.985		*0.984*		**0.147**		0.415		0.780	*0.821*	*1.525*	*14.393*
	DTM	*0.984*		0.991		**0.068**		0.492		*0.760*	*0.817*	*1.354*	11.769
	DPT	**0.986**		**0.992**		**0.245**		0.408		0.759	0.816	1.346	12.316
**(b) Dataset 2: Four classes**													
**Loss** **Function**	**Weighting**	**Cerebrum**				**Cerebellum**				**Blood Vessels**			
		**DSC**	**SDSC**	**ASD**	**95HD**	**DSC**	**SDSC**	**ASD**	**95HD**	**DSC**	**SDSC**	**ASD**	**95HD**
Cross entropy	N/A	0.985	0.994	0.057	0.469	0.978	0.981	0.082	0.670	0.792	0.834	1.209	11.215
	Inverse	*0.966*	*0.963*	*0.221*	*1.015*	*0.939*	*0.890*	*0.472*	*1.911*	*0.623*	*0.668*	*2.375*	*19.928*
	Median	*0.970*	*0.968*	*0.221*	*1.009*	*0.954*	*0.938*	*0.279*	*1.397*	*0.674*	*0.738*	*1.860*	*17.051*
	Focal	*0.980*	*0.990*	*0.087*	*0.575*	0.979	0.982	0.082	0.635	0.783	0.836	1.168	11.228
	DTM	**0.986**	0.994	0.059	**0.408**	0.977	0.979	0.142	2.019	*0.781*	0.827	1.247	11.639
	DPT	*0.982*	*0.992*	*0.069*	*0.505*	**0.980**	**0.986**	**0.065**	0.579	0.791	0.842	1.138	11.197
Dice	N/A	0.986	0.993	0.060	0.338	0.975	0.971	0.329	2.370	0.766	0.821	1.246	11.110
	Inverse	*0.163*	*0.066*	*18.575*	*81.644*	*0.960*	*0.949*	0.314	*3.939*	**0.799**	**0.840**	1.192	12.014
	Median	*0.980*	*0.984*	*0.155*	*0.524*	0.973	0.972	**0.234**	2.578	**0.780**	0.818	1.306	12.029
	Focal	0.987	0.994	**0.052**	0.352	**0.980**	**0.986**	**0.067**	**0.543**	**0.791**	0.834	1.233	11.518
	DTM	*0.971*	*0.963*	*0.198*	*1.061*	*0.956*	*0.933*	0.449	*3.654*	*0.610*	*0.630*	*5.309*	*34.425*
	DPT	*0.985*	*0.992*	0.064	*0.505*	0.978	0.981	**0.085**	0.593	**0.786**	0.827	1.289	12.360
**(c) Dataset 3: Five classes**													
**Loss** **Function**	**Weighting**	**Cerebrum**								**Cerebellum**			
		**DSC**		**SDSC**		**ASD**		**95HD**		**DSC**	**SDSC**	**ASD**	**95HD**
Cross entropy	N/A	0.981		0.991		0.083		0.552		0.977	0.980	0.127	0.855
	Inverse	*0.971*		*0.973*		*0.179*		*0.846*		*0.950*	*0.926*	*0.346*	*1.492*
	Median	*0.979*		*0.987*		*0.104*		*0.609*		*0.958*	*0.949*	*0.253*	*1.252*
	Focal	**0.985**		**0.993**		**0.060**		**0.469**		0.979	0.984	0.107	0.634
	DTM	*0.980*		0.990		0.085		0.552		0.979	0.982	0.093	0.898
	DPT	**0.982**		**0.993**		**0.069**		**0.502**		0.980	0.985	0.070	0.624
Dice	N/A	0.986		0.993		0.074		0.338		0.977	0.982	0.084	0.618
	Inverse	*0.000*		*0.000*		-		-		*0.955*	*0.946*	*0.221*	*1.405*
	Median	*0.984*		*0.988*		*0.107*		*0.502*		*0.974*	*0.975*	*0.171*	1.164
	Focal	**0.987**		**0.995**		**0.052**		0.291		**0.980**	**0.986**	**0.065**	0.567
	DTM	0.986		0.993		**0.068**		0.361		0.978	0.983	**0.082**	0.608
	DPT	*0.985*		*0.992*		*0.098*		*0.445*		0.974	0.977	0.095	0.747
**Loss** **Function**	**Weighting**	**Brainstem**								**Blood Vessels**			
		**DSC**		**SDSC**		**ASD**		**95HD**		**DSC**	**SDSC**	**ASD**	**95HD**
Cross entropy	N/A	0.961		0.942		0.266		2.083		0.790	0.846	1.084	10.471
	Inverse	*0.944*		0.937		*0.371*		1.302		*0.712*	*0.778*	*1.524*	*14.184*
	Median	*0.949*		*0.928*		*0.415*		1.528		*0.686*	*0.721*	*1.920*	*17.233*
	Focal	0.962		0.947		0.267		1.495		0.782	*0.830*	*1.263*	12.068
	DTM	**0.966**		0.946		0.291		2.362		*0.783*	*0.840*	*1.163*	*11.097*
	DPT	**0.964**		**0.952**		0.203		**1.343**		**0.797**	**0.855**	1.059	10.703
Dice	N/A	0.960		0.934		0.389		2.174		0.774	0.828	1.234	11.574
	Inverse	0.961		0.941		0.391		2.374		**0.801**	0.836	1.196	12.002
	Median	0.962		0.941		0.344		2.329		**0.788**	0.829	1.200	10.648
	Focal	**0.963**		**0.952**		**0.235**		**1.262**		0.783	0.828	1.300	12.835
	DTM	**0.964**		**0.944**		**0.217**		**1.288**		0.773	0.831	1.221	11.280
	DPT	0.960		0.929		0.394		3.759		*0.757*	*0.801*	*1.869*	*18.269*

**Table 6:** Ranking results of no weighting (N/A), inverse frequency weighting (Inverse), inverse median frequency weighting (Median), focal weighting (Focal), distance transform map-based weighting (DTM), and distance penalty term-based weighting (DPT) in (a) binary-class segmentation tasks and (b) multi-class segmentation tasks. The best results are shown in bold. The rank is determined based on the rank scores of segmentation results on all datasets.

(a) Binary-Class Segmentation Tasks							
Loss Function	Weighting	Rank Score					Rank
		Dataset 1: Cerebrum	Dataset 2: Cerebellum	Dataset 3: Brainstem	Dataset 4: Blood Vessels	All	
Cross entropy	N/A	5.25	**7.25**	3.25	**6.00**	5.44	4
	Inverse	0.00	2.25	1.25	1.25	1.19	11
	Median	1.50	0.75	0.50	0.75	0.88	12
	Focal	3.50	4.00	6.00	**6.00**	4.88	5
	DTM	4.25	6.25	**6.50**	**6.00**	5.75	2
	DPT	5.5	6.25	4.50	**6.00**	5.56	3
Dice	N/A	2.75	4.00	0.00	2.50	2.31	10
	Inverse	1.75	1.50	3.00	5.50	2.94	8
	Median	1.75	1.00	3.50	3.75	2.50	9
	Focal	**8.5**	4.50	**6.50**	4.75	**6.06**	1
	DTM	4.5	4.25	4.25	1.75	3.69	6
	DPT	5.25	4.00	4.00	0.00	3.31	7
**(b) Multi-class segmentation tasks**							
**Loss** **Function**	**Weighting**	**Rank Score**					**Rank**
		**Dataset 1: Three Classes**	**Dataset 2: Four Classes**	**Dataset 3: Five Classes**		**All**	
Cross entropy	N/A	1.50	5.75	4.13		4.08	6
	Inverse	0.63	0.83	0.81		0.78	12
	Median	1.25	1.92	0.81		1.28	11
	Focal	4.88	4.67	4.19		4.50	4
	DTM	5.63	5.25	3.69		4.64	3
	DPT	**6.75**	6.17	6.63		**6.50**	1
Dice	N/A	4.63	4.58	3.69		4.19	5
	Inverse	2.88	2.17	1.38		1.97	10
	Median	6.00	3.67	2.56		3.69	8
	Focal	3.63	**7.50**	**6.75**		6.31	2
	DTM	4.63	0.67	4.75		3.36	9
	DPT	4.88	4.67	2.44		3.72	7

## Data Availability

Data sharing not applicable.

## References

[1] González-Villà S., Oliver A., Valverde S., Wang L., Zwiggelaar R., Lladó X. (2016). A review on brain structures segmentation in magnetic resonance imaging. Artif. Intell. Med..

[2] Despotovic I., Goossens B., Philips W. (2015). MRI segmentation of the human brain: Challenges, methods, and applications. Comput. Math. Methods Med..

[3] Long J., Shelhamer E., Darrell T. (2015). Fully convolutional networks for semantic segmentation. Proceedings of the IEEE Conference on Comput Vision and Pattern Recognition.

[4] Ronneberger O., Fischer P., Brox T. U-net: Convolutional networks for biomedical image segmentation. Proceedings of the International Conference on Medical Image Computing and Computer-Assisted Intervention.

[5] Çiçek Ö., Abdulkadir A., Lienkamp S.S., Brox T., Ronneberger O. 3D U-Net: Learning dense volumetric segmentation from sparse annotation. Proceedings of the International Conference on Medical Image Computing and Computer-Assisted Intervention.

[6] Bernal J., Kushibar K., Asfaw D.S., Valverde S., Oliver A., Marti R., Lladó X. (2019). Deep convolutional neural networks for brain image analysis networks for brain image analysis on magnetic resonance imaging: A review. Artif. Intell. Med..

[7] Buda M., Maki A., Mazuroqski M.A. (2018). A systematic study of the class imbalance problem in convolutional neural networks. Neural Netw..

[8] Zhou T., Ruan S., Canu S. (2019). A review: Deep learning for medical image segmentation using multi-modality fusion. Array.

[9] Jang J., Eo T.J., Kim M., Choi N., Han D., Kim D., Hwang D. Medical image matching using variable randomized undersampling probability pattern in data acquisition. Proceedings of the 2014 International Conference on Electronics, Information and Communications.

[10] Chawla N.V., Bowyer K.W., Hall L.O., Kegelmeyer W.P. (2002). SMOTE: Synthetic minority over-sampling technique. J. Artif. Intell. Res..

[11] Milletari F., Navab N., Ahmadi S.A. (2016). V-net: Fully convolutional neural networks for volumetric medical image segmentation. Proceedings of the Fourth International Conference on 3D Vision.

[12] Drozdzal M., Vorontsov E., Chartrand G., Kadoury S., Pal C. (2016). The importance of skip connections in biomedical image segmentation. Deep Learning and Data Labeling for Medical Applications.

[13] Rahman M.A., Wang Y. Optimizing intersection-over-union in deep neural networks for image segmentation. Proceedings of the International Symposium on Visual Computing.

[14] Berman M., Triki A.R., Blaschko M.B. The lovász-softmax loss: A tractable surrogate for the optimization of the intersection-over-union measure in neural networks. Proceedings of the IEEE Conference on Computer Vision and Pattern Recognition.

[15] Wong K.C.L., Moradi M., Tang H., Syeda-Mahmood T. 3D segmentation with exponential logarithmic loss for highly unbalanced object sizes. Proceedings of the International Conference on Medical Image Computing and Computer-Assisted Intervention.

[16] Kervadec H., Bouchtiba J., Desrosiers C., Granger E., Dolz J., Ayed I.B. (2019). Boundary loss for highly unbalanced segmentation. Med. Image Anal..

[17] Karimi D., Salcudean S.E. (2020). Reducing the Hausdorff distance in medical image segmentation with convolutional neural networks. IEEE Trans. Med. Imaging.

[18] Eigen D., Fergus R. (2015). Predicting depth, surface normal and semantic labels with a common multi-scale convolutional architecture. Proceedings of the IEEE International Conference on Computer Vision.

[19] Lin T.Y., Goyal P., Girshick R., He K., Dollár P. (2017). Focal loss for dense object detection. Proceedings of the IEEE International Conference on Computer Vision.

[20] Caliva F., Iriondo C., Martinez A.M., Majumdar S., Pedoia V. Distance map loss penalty term for semantic segmentation. Proceedings of the 2nd International Conference on Medical Imaging with Deep Learning.

[21] Salehi S.S.M., Erdogmus D., Gholipour A. Tversky loss function for image segmentation using 3D fully convolutional deep networks. Proceedings of the International Workshop on Machine Learning in Medical Imaging.

[22] Hashemi S.R., Salehi S.S.M., Erdogmus D., Prabhu S.P., Warfield S.K., Gholipour A. (2018). Asymmetric loss functions and deep densely-connected networks for highly-imbalanced medical image segmentation: Application to multiple sclerosis lesion detection. IEEE Access.

[23] Guerrero-Pena F.A., Fernandez P.D.M., Ren T.I., Yui M., Rothenberg E., Cunha A. Multiclass weighted loss for instance segmentation of cluttered cells. Proceedings of the 25th IEEE International Conference on Image Processing.

[24] Sudre C.H., Li W., Vercauteren T., Ourselin S., Cardoso M.J. (2017). Generalised Dice overlap as a deep learning loss function for highly unbalanced segmentations. Proceedings of the Deep Learning in Medical Image Analysis and Multimodal Learning for Clinical Decision Support.

[25] Li X., Sun X., Meng Y., Liang J., Wu F., Li J. (2020). Dice loss for data-imbalanced NLP tasks. Proceedings of the 58th Annual Meeting of the Association for Computational Linguistics.

[26] Ma J., Chen J., Ng M., Huang R., Li Y., Li C., Yang X., Martel A.L. (2021). Loss odyssey in medical image segmentation. Med. Image Anal..

[27] Ma J., Wei Z., Zhang Y., Wang Y., Lv R., Zhu C., Chen G., Liu J., Peng C., Wang L. (2020). How distance transform maps boost segmentation CNNs: An empirical study. Med. Imaging Deep Learn..

[28] Yeung M., Sala E., Schönlieb C.B., Rundo L. (2021). Unified Focal loss: Generalising Dice and cross entropy-based losses to handle class imbalanced medical image segmentation. arXiv.

[29] Huo Y., Xu Z., Xiong Y., Aboud K., Parvathaneni P., Bao S., Bermudez C., Resnick S.M., Cutting L.E., Landman B.A. (2019). 3D whole brain segmentation using spatially localized atlas network tiles. NeuroImage.

[30] Taghanaki S.A., Zheng Y., Zhou S.K., Georgescu B., Sharma P., Xu D., Comaniciu D., Hamarneh G. (2019). Combo loss: Handling input and output imbalance in multi-organ segmentation. Comput. Med. Imaging Graph..

[31] Zhu W., Huang Y., Zeng L., Chen X., Liu Y., Qian Z., Du N., Fan W., Xie X. (2018). AnatomyNet: Deep learning for fast and fully automated whole-volume segmentation of head and neck anatomy. Med. Phys..

[32] Xue Y., Tang H., Qiao Z., Gong G., Yin Y., Qian Z., Huang X. (2020). Shape-aware organ segmentation by predicting signed distance maps. AAAI Conf. Artif. Intell..

[33] Kingma D.P., Ba J.L. (2014). Adam: A method for stochastic optimization. arXiv.

[34] Nikolov S., Blackwell S., Zverovitch A., Mendes R., Livne M., De Fauw J., Patel Y., Meyer C., Askham H., Romera-Paredes B. (2018). Deep learning to achieve clinically applicable segmentation of head and neck anatomy for radiotherapy. arXiv.

[35] DeepMind Github: Library to Compute Surface Distance Based Performance Metrics for Segmentation Tasks. https://github.com/deepmind/surface-distance.

[36] Antonelli M., Reinke A., Bakas S., Farahani K., Kopp-Schneider A., Landman B.A., Litjens G., Menze B., Ronneberger O., Summers R.M. (2021). The Medical Segmentation Decathlon. arXiv.

[37] Conti V., Militello C., Rundo L., Vitabile S. (2020). A novel bio-inspired approach for high-performance management in service-oriented networks. IEEE Trans. Emerg. Top. Comput..

